# Audiologic monitoring of multi-drug resistant tuberculosis patients on aminoglycoside treatment with long term follow-up

**DOI:** 10.1186/1472-6815-7-5

**Published:** 2007-11-12

**Authors:** Prahlad Duggal, Malay Sarkar

**Affiliations:** 1 Department of Otolaryngology, Dr. Rajinder Prasad Govt. Medical College, Tanda, Kangra, Himachal Pradesh, India; 2 Department of Pulmonary Medicine, Dr. Rajinder Prasad Govt. Medical College, Tanda, Kangra, Himachal Pradesh, India

## Abstract

**Background:**

Multi-drug resistant tuberculosis has emerged as a significant problem with the resurfacing of tuberculosis and thus the need to use the second line drugs with the resultant increased incidence of adverse effects. We discuss the effect of second line aminoglycoside anti-tubercular drugs on the hearing status of MDR-TB patients.

**Methods:**

Sixty four patients were put on second line aminoglycoside anti-TB drugs. These were divided into three groups: group I, 34 patients using amikacin, group II, 26 patients using kanamycin and group III, 4 patients using capreomycin.

**Results:**

Of these, 18.75% of the patients developed sensorineural hearing loss involving higher frequencies while 6.25% had involvement of speech frequencies also. All patients were seen again approximately one year after aminoglycoside discontinuation and all hearing losses were permanent with no threshold improvement.

**Conclusion:**

Aminoglycosides used in MDR-TB patients may result in irreversible hearing loss involving higher frequencies and can become a hearing handicap as speech frequencies are also involved in some of the patients thus underlining the need for regular audiologic evaluation in patients of MDR-TB during the treatment.

## Background

Tuberculosis is one of the leading infectious diseases in the world and is responsible for more than two million deaths and nine million new cases annually [1]. Emergence of resistance to drugs used to treat tuberculosis and particularly multi-drug resistant (MDR-TB) has become an obstacle to effective global TB control [2]. Incomplete and inadequate treatment is the most important factor leading to its development, suggesting that it is often a man-made problem [3]. Inappropriate treatment results in unacceptably low cure rates and the continued spread of tuberculosis in the community because of selection of M. tuberculosis isolates that are resistant to anti- tubercular drugs [4]. Taking into consideration the high success rate of TB treatment under DOT policy (Directly Observed Treatment), the principal cause for the generation of drug resistant TB generally appears to lie in the low degree of patient compliance with treatment [5]. Most of the problems from which drug-resistance originates are related to the length of treatment (especially considering tolerability and adherence), the longer time that is required to treat MDR-TB results in an additional risk of poor treatment adherence and thus of treatment failure [6]. Two other major issues of importance which affect the outcome in MDR-TB compared to drug-susceptible disease are the increased cost (up to 100 times higher) and higher toxicity [7,8].

By definition, chemotherapy of MDR-TB cannot rely upon isoniazid and rifampicin, the two most powerful drugs for the treatment of tuberculosis [9]. Thus, depending on the individual susceptibility pattern, residual first-line oral drugs must be appropriately combined with additional second line drugs comprising injectable aminoglycosides (amikacin, kanamycin, capreomycin), fluoroquinolones (ciprofloxacin, ofloxacin, levofloxacin, moxifloxacin, gatifloxacin), old bacteriostatic second line anti-tuberculosis agents (ethionamide, protionamide, cycloserine, para-amino salicylic acid, thiocetazone) and anti-tuberculosis agents with unclear efficacy (clofazimine, amoxicillin/clavuanate, clarithromycin, linezolid) [2].

A crucial issue related to long-term administration of the injectable group is toxicity. Ototoxicity and nephrotoxicity are well recognized as dose-related adverse effects of aminoglycosides [10]. Ototoxicity and nephrotoxicity have been of major concern because of the narrow therapeutic range of these agents and the wide variability in pharmacokinetics among patients [11]. Amikacin is a semi-synthetic aminoglycoside and shows excellent activity against Mycobacterium tuberculosis and atypical mycobacteria and has been used in the treatment of disseminated atypical Mycobacterium infection in AIDS patients [12].

Kanamycin, an antibiotic elaborated by Streptomyces kanamyceticus has shown activity against Mycobacterium tuberculosis. But as the therapy of this disease is protracted and involves the administration of large total doses of the drug, with the risk of ototoxicity and nephrotoxicity, kanamycin should be used only in infection with organisms that are resistant to the more commonly used agents [12]. It is more toxic to cochlea with well documented ototoxicity [13,14] but is still being commonly used in clinical settings like ours (in developing countries) for MDR-TB where cost considerations are a major factor in patient compliance (because of having one fourth the cost of amikacin and one tenth the cost of capreomycin).

Capreomycin is an antimicrobial cyclic peptide elaborated by Streptomyces capreolus and is effective both in vitro and in experimental tuberculosis. It has proven to be of value in the therapy of 'resistant' or treatment failure tuberculosis when given with ethambutol or isoniazid [15]. The toxicity profile of capreomycin is similar to that of aminoglycosides and has been discussed along with aminoglycosides in the present study [16]. Cost of therapy with capreomycin is quite high compared to amikacin and kanamycin and is used only in few patients of MDR-TB showing resistance to amikacin and kanamycin. Initial ototoxic drug exposure typically affects cochlear regions coding the high frequencies. Continued exposure results in spread of damage to progressively lower frequencies. Early identification of ototoxic hearing loss provides physicians the opportunity to adjust the therapeutic treatment in order to minimize or prevent hearing loss requiring rehabilitation, depending on a patient's overall treatment picture [17,18]. The present study was conducted to study the effect of second line aminoglycosides (amikacin, kanamycin and capreomycin) on the hearing status in patients of MDR-TB after long term use as a part of multi-drug therapy.

## Methods

A total of 64 patients were included in the study who completed treatment for MDR-TB from April 2000 to September 2006 using second line drugs and fulfilled the criteria described below. Treatment regimens followed were based on drug susceptibility testing and previous treatment history. All the relevant data were recorded on MDR-TB patient sheets including the baseline and follow-up investigations. Baseline pre-treatment pure tone audiometry was performed on all the patients and repeated every two months until completion of therapy. Other baseline investigations including renal function and liver function tests were also performed on all the patients. These were not repeated during the therapy unless some specific complaint or clinical suspicion was present. Patients with abnormal pre-treatment renal functions were excluded from the study group. Serum aminoglycosides levels were not measured during the course of therapy as the costs involved did not permit them. Repeat renal function tests were performed in all the patients with complaint/audiologic evidence of hearing loss after start of aminoglycoside treatment. Those patients with any abnormality in repeated renal function tests were excluded from the study and were managed in consultation with the medical specialist. The treatment regimens used were in accordance with the standard recommended protocols with necessary permissions and informed consent of all the patients in the study was obtained.

Complete otolaryngologic examination was done as a part of pre-treatment clinical examination in all the patients. Those patients with any pre-treatment evidence of hearing loss on history, clinical assessment (patients with evidence of infective pathology in ear were excluded) or pure tone audiometry, whether conductive (A-B gap > 10 dB) or sensorineural were excluded. Baseline pure tone audiograms between 125 Hz and 8000 Hz were performed for all the patients in a sound treated room. Baseline, in addition to follow-up audiological testing, included both air-and bone-conduction threshold measurements in all the patients. All the patients who had received any ototoxic drug as a part of previous regimen were excluded from the study.

The criteria used for determining ototoxic threshold shift from baseline audiogram were: (1) 20 dB or greater decrease at any one test frequency, (2) 10 dB or greater decrease at any two adjacent frequencies, or (3) loss of response at three consecutive frequencies where responses were previously obtained (ASHA, 1994)[19]. All the changes were confirmed by retest on the same day. These patients were divided into three groups depending upon the ototoxic aminoglycoside used. Group I (n = 34) patients received amikacin (15 mg/kg per day, intramuscular, single daily dose), group II (n = 26) patients received kanamycin (15 mg/kg per day, intramuscular, single daily dose) and group III (n = 4) patients received capreomycin (15 mg/kg per day, intramuscular, single daily dose) as a part of complete regimen comprising of one injectable (aminoglycoside), one quinolone with a minimum of five drugs depending on the drug sensitivity testing and the costs involved. Streptomycin was not used because of high resistance in these patients on drug sensitivity testing. Those patients receiving two ototoxic drugs were excluded from the study. Total duration of therapy in all the patients ranged from 18 to 24 months after sputum smear/culture conversion and aminoglycoside was used for six months after sputum conversion.

Audiometry findings were considered under three categories; 'normal' (N) defined by patients with pure tone audiograms showing air-conduction thresholds up to 20 ± 5 dB HL at all the tested frequencies from 125 Hz to 8000 Hz with air-bone gap of ≤ 10 dB; 'high frequency loss' (HFL) defined by (1) a 20 dB or greater decrease at any of the three frequencies; 4000, 6000 and 8000 Hz, (2) 10 dB or greater decrease at any two adjacent frequencies in above range, (3) loss of response at all the three frequencies (4000, 6000 and 8000 Hz) where responses were previously obtained; and 'flat' (FLAT) when in addition to HFL, above criteria were also fulfilled in the frequencies ranging from 250 to 3000 Hz. All the pure tone threshold shifts were recorded at each frequency tested with reference to the baseline pure tone threshold at the same frequency. Ototoxic aminoglycoside was stopped in patients complaining of or showing audiological evidence of hearing loss (based on ASHA's criteria used for determining ototoxic threshold shift, 1994) and substituted with other second line drug/drugs depending upon the drug sensitivity testing. The data so obtained was analyzed for various epidemiological factors and hearing status in each group after completion of therapy and data were reported as mean and standard deviations.

## Results

All the patients in this study were in the age group of 17 to 65 years (mean age = 39.9 ± 13.5 years) with males constituting 60.9% and females constituting 39.1% (n = 64). Majority of the patients were from rural background (68.7%) while 31.2% were from urban areas (n = 64). Majority (65.6%) of the patients were from low socio-economic status (n = 64). Overall incidence of HFL was 18.75% while incidence of FLAT loss was 6.25% in the present study (n = 64). The mean duration of therapy was 20.3 ± 0.25 months after smear/culture conversion (range was 18–24 months) while aminoglycosides were continued for 6 months (180 days) post conversion in the initial phase. Total duration of aminoglycoside use was 233.3 + 106.6 days while duration of audiologic follow-up after discontinuation of aminoglycoside use was 376.7 ± 42 days.

Seven patients (20.6%, n = 34) of group I (amikacin) showed sensorineural hearing loss (SNHL) involving the higher frequencies (HFL). Amikacin was stopped on the first report of hearing loss and patient shifted to another of the second line drug. Follow-up audiogram showed development of FLAT loss in two (5.9%, n = 34) of these patients, (Subject # 2) at six months (pure tone audiogram at 6 months, PTA6) and (Subject # 4) at 10 months (pure tone audiogram at 10 months, PTA10) (Table 1). Duration of aminoglycoside use in group I was 235 ± 40 days (n = 34) while duration of aminoglycoside use in seven patients of group I which developed ototoxicity (n = 7) was 163 ± 45 days (as amikacin was discontinued on first report of hearing loss).

**Table 1:** Audiometry findings, present regimen and length of aminoglycoside use in group I(n = 7) and II (n = 4) patients showing audiological evidence of hearing loss

Sr. No. (Group)	Length of treatment (Days)	Present regimen (Drugs)	PTA0	PTA2	PTA4	PTA6	PTA8	PTA10	PTAc
1 (I)	180	A, F, E, P, Py	N	N	N	HFL	HFL	HFL	HFL
2 (I)	60	A, F, E, P, Py	N	HFL	HFL	HFL	FLAT	FLAT	FLAT
3 (I)	180	A, F, E, P, Py	N	N	N	HFL	HFL	HFL	HFL
4 (I)	180	A, F, E, P, Py	N	N	N	HFL	HFL	FLAT	FLAT
5 (I)	180	A, F, E, P, Py	N	N	N	HFL	HFL	HFL	HFL
6 (I)	180	A, F, E, P, Py	N	N	N	HFL	HFL	HFL	HFL
7 (I)	180	A, F, E, P, Py	N	N	N	HFL	HFL	HFL	HFL
8 (II)	120	K, F, E, P, Py	N	N	N	HFL	FLAT	FLAT	FLAT
9 (II)	180	K, F, E, P, Py	N	N	N	HFL	HFL	HFL	HFL
10 (II)	180	K, F, E, P, Py	N	N	N	HFL	HFL	FLAT	FLAT
11 (II)	180	K, F, E, P, Py	N	N	N	HFL	HFL	HFL	HFL

A = amikacin, F = flouroquinolone, E = ethionamide, P = Para-amino salicylic acid, Py = Pyrazinamide, K = kanamycin, Days = days of aminoglycoside use in present regimen, PTA0 = baseline pure tone audiogram, PTA2 = pure tone audiogram after 2 months of aminoglycoside use, PTA4 = pure tone audiogram after 4 months of aminoglycoside use, PTA6 = pure tone audiogram after 6 months of aminoglycoside use, PTA8 = pure tone audiogram after 8 months of aminoglycoside use, PTA10 = pure tone audiogram after 10 months of aminoglycoside use, PTAc = pure tone audiogram after completion of therapy for MDR-TB, N = Normal, HFL = High frequency loss (hearing loss involving frequencies of 4000, 6000 and 8000 Hz), FLAT = Hearing loss involving frequencies in range of 250–3000 Hz along with involvement of 4000, 6000 and 8000 Hz (Criteria for hearing loss as defined in methods section).

Four patients (15.4%, n = 26) of group II (kanamycin) had SNHL involving higher frequencies (HFL). In two (7.7%, n = 26) patients lower frequencies were also involved (FLAT) even when Subject # 8 had injectable drug stopped at 4 months while Subject # 10 had the drug stopped on report of high frequency loss at 6 months (Table 1). Duration of aminoglycoside use in group II was 237 ± 34 days (n = 26) while duration of aminoglycoside use in four patients of group II which developed ototoxicity (n = 4) was 165 ± 30 days (as kanamycin was discontinued on first report of hearing loss).

One of the patients of group III (25.0%, n = 4) developed sensorineural hearing loss involving high frequencies (HFL) at 4 months (Table 2) and capreomycin was substituted with other second line drug based on drug sensitivity testing. Mean duration of aminoglycoside use in group III was 215 ± 64 (n = 4) days. Duration of capreomycin use in single patient with HFL (Subject # 12) was 120 days. None of the patients had any recovery in pure-tone thresholds after stopping the injectable treatment. Group-wise mean loss shown by patients with ototoxic threshold shift (Group I, n = 7; Group II, n = 4, Group III, n = 1) at different frequencies tested in the present study is shown in Table 3. Mean loss (average decrease in pure tone threshold from baseline at each frequency tested in patients with ototoxic threshold shift in each group) in seven patients of group I fulfilled the criteria (ASHA' 1994) for ototoxic threshold shift in frequencies range of 4000–8000 Hz (HFL) but not in the frequency range from 250–3000 Hz. Mean loss in four patients of group II fulfilled the criteria for ototoxic threshold shift in both the frequency ranges i.e. 4000–8000 Hz (HFL) and 250–3000 Hz. As sample size for group III was small, the data was not further analyzed but a note of ototoxic threshold shift in 4000–8000 Hz range was made. Studies with larger number of patients using capreomycin are required but higher cost of the therapy with capreomycin limits its use in large number of patients in our setting.

**Table 2:** Audiometry findings, present regimen and length of aminoglycoside use in all four patients using capreomycin (Group III)

Sr. No.	Length of treatment (Days)	Present regimen (Drugs)	PTA0	PTA2	PTA4	PTA6	PTA8	PTA10	PTAc
1	247	C, F, E, P, Clo	N	N	N	N	N	N	N
2	254	C, F, E, P, Clo	N	N	N	N	N	N	N
3*	120	C, F, E, P, Clo	N	N	HFL	HFL	HFL	HFL	HFL
4	240	C, F, E, P, Clo	N	N	N	N	N	N	N

C = capreomycin, F = flouroquinolone, E = ethionamide, P = Paraminosalicylic acid, Clo = Clofazimine, Days = days of aminoglycoside use in present regimen, PTA0 = baseline pure tone audiogram, PTA2 = pure tone audiogram after 2 months of aminoglycoside use, PTA4 = pure tone audiogram after 4 months of aminoglycoside use, PTA6 = pure tone audiogram after 6 months of aminoglycoside use, PTA8 = pure tone audiogram after 8 months of aminoglycoside use, PTA10 = pure tone audiogram after 10 months of aminoglycoside use, PTAc = pure tone audiogram after completion of therapy for MDR-TB, N = Normal, HFL = High frequency loss (hearing loss involving frequencies of 4000, 6000 and 8000 Hz), FLAT = Hearing loss involving frequencies in range of 250–3000 Hz along with involvement of 4000, 6000 and 8000 Hz (Criteria for hearing loss as defined in methods section). * Patient in group III with audiological evidence of hearing loss

**Table 3:** Mean loss in patients with ototoxic threshold shift in group I (n = 7), group II (n = 4) and group III (n = 1) at different frequencies tested

Group	250 Hz	500 Hz	1000 Hz	2000 Hz	3000 Hz	4000 Hz	6000 Hz	8000 Hz
I	3 ± 5.7	2 ± 5.7	3 ± 3.8	5 ± 5	8 ± 8.6	17 ± 12.5	29.3 ± 9.3	34.3 ± 8.5
II	2.5 ± 5	5 ± 7.1	10 ± 9.1	16.25 ± 10.3	20 ± 14.7	27.5 ± 10.4	30 ± 7.9	33.75 ± 16
III	-	-	5	5	10	30	35	45

## Discussion

MDR-TB is a growing problem throughout the world [20]. The selection of drug resistant M. tuberculosis depends on the frequency of the specific drug-resistant mutants in the initially drug-susceptible bacterial population. As a consequence, the chance of selecting such mutants is highest in the case of mono-therapy [4] and this is the rationale of combination chemotherapy both in case of drug-susceptible as well as MDR-TB even at the cost of adverse drug reactions so that mutants resistant to a single drug are not easily selected by mono-therapy. Adherence to treatment is a critical factor in the management of MDR-TB and adverse events associated with second line drugs could have a severe impact on adherence because long term use of second line drugs is required in MDR-TB ranging from 18–24 months [21]. A large literature exists on the adverse effects of anti-tuberculosis medications, which range from minor to life threatening [10,22-24].

The main constraint to the administration of aminoglycosides are risks of nephrotoxicity and ototoxicity [24]. Ototoxicity is the major irreversible toxicity of aminoglycosides. Cochlear damage can produce permanent hearing loss, while damage to vestibular apparatus results in dizziness, ataxia and/or nystagmus. Aminoglycosides appear to generate free radicals within the inner ear, with subsequent permanent damage to sensory cells and neurons resulting in permanent hearing loss [14]. The other major limitation to the clinical use of aminoglycosides continues to be concern for the development of nephrotoxicity. Nephrotoxicity has been defined as an increase in the baseline serum creatinine concentration of 0.5 mg/dl or a 50% increase, whichever is greater, on two consecutive occasions any time during therapy or up to 1 week after the cessation of therapy [25]. Evidence from studies with animals and humans has demonstrated a correlation between the nephrotoxic effect of aminoglycosides and the accumulation of these drugs in the cortex of kidney [26-28].

The present study evaluates the effect of parenteral second line aminoglycosides namely amikacin, kanamycin and capreomycin on hearing status of MDR-TB patients. We report a hearing loss documented by pure tone audiometry in 18.75% patients of MDR-TB using a single parenteral second line aminoglycoside involving higher frequencies (4000 to 8000 Hz) to start with and progressing to involve lower frequencies (500, 1000, 2000 and 3000 Hz) in 6.25% thus affecting the speech comprehension of the patient (n = 64). Speech comprehension can also be affected with hearing loss in the 4000 Hz range and may adversely affect communication especially in situations like environments with back ground noise [29]. The loss once developed has been found to be irreversible and none of the patients in the present study showed any improvement after stopping the therapy.

Ototoxicity is determined by comparing baseline data, ideally obtained prior to ototoxic drug administration, to the results of subsequent monitoring tests. Detecting changes in pure tone thresholds directly using serial audiograms is considered the most effective indicator of ototoxic hearing loss, particularly when ultra-high frequency thresholds are included [17,18]. Monitoring audiological evaluations after the baseline evaluations have been recommended 1–2 times per week for patients receiving ototoxic antibiotics [17,18,25]. Other approaches to audiologic monitoring for ototoxicity are high frequency audiometry and otoacoustic emissions [30].

In the present study, pure tone audiometry was performed every other month for each patient until the completion of therapy. Because aminoglycoside ototoxicity can progress after discontinuation of the drug [13], we also performed audiometric follow-up in all patients for an average of over one year after drug discontinuation. This long term follow-up confirmed that all aminoglycoside-induced hearing loss in this patient population was permanent and not reversible. Persistence of toxicity of sera has been reported up to one year in patients using aminoglycosides even after stopping the ototoxic drug [31]. Twice weekly audiograms as recommended were not performed in the present study because of cost involved and the inability of the patients from far distant places to report twice weekly at our center where facilities for conventional assessment of hearing are available. It is not common to find equipment for audiometry as well as trained staff at peripheral centers in developing country like ours. Conventional frequency range (250–8000 Hz) was used in the present study as only conventional audiometers with frequency range between 125 and 8000 Hz are available with us owing to low cost compared to high frequency equipment.

Different studies have reported hearing loss as an adverse drug reaction in patients of MDR-TB ranging from 6–18% [21,22,31]. The finding that higher frequencies are involved before the lower frequencies may be used as a monitoring procedure for the detection of ototoxicity and has the potential for minimizing irreversible communication deficits in patients receiving aminoglycoside therapy [32]. In all the patients showing hearing loss, the aminoglycoside was stopped and changed to another second line drug done. Incidence of hearing loss may have been reduced because the aminoglycoside was stopped immediately at the outset of ototoxicity and substituted with another second line drug. All the patients included in the present study completed the remaining part of the therapy. Other authors have also reported changing to other second line drugs and completion of full therapy [21,22].

A number of otoprotective agents are being investigated for protection against hearing loss induced by cisplatin, carboplatin, aminoglycosides or noise exposure. These agents delivered either before or in combination with ototoxic drugs may help to prevent ototoxicity. D-methionine as an otoprotective agent has shown protection against amikacin induced ototoxicity [33].

There is evidence that aminoglycoside accumulation in the kidney may be related to the dosing schedule i.e. administration of larger doses less frequently may reduce the level of drug accumulation in the kidney and has been prospectively shown to reduce the nephrotoxic potentials of aminoglycosides [25]. Conventional multiple daily dosing is being gradually abandoned in favor of once daily dosing and results from meta-analysis of randomized clinical trials show diminished [34] or comparable [35-38] nephrotoxicity, better [35,36,39] or comparable [34,37,38] efficacy and comparable [34-36,38,39] ototoxicity with once daily dosing among adults. Once daily dosing has been used in all the patients in the present study but individualized dosing based on monitoring of serum levels of aminoglycosides has not been used in present study.

Individualized aminoglycoside dosing guided by targeted peak and trough concentrations in serum on the basis of the patient's individual pharmacokinetics parameters and standard equations has been related to decreased toxicity [40]. An association of ototoxicity with nephrotoxicity and with an elevated mean trough aminoglycoside serum level has been observed in patients treated with aminoglycosides [41]. Because of economic constraints and the non-affordability by the patients, serum levels of aminoglycosides during the therapy were not measured in the present study. But with individualized dosing based on patient's individual pharmacokinetic monitoring guided by targeted peak and trough concentrations, side effects could probably be avoided in some cases.

First row outer hair cells (OHCs) in the basal turn tend to be affected earlier than inner apical cells and type I cells are affected before type II cells. The progression of hair cell loss in cochlea tends to be from basal to apical and from OHCs to inner hair cells (IHCs) to supporting cells to more central neural structures like spiral ganglion cells [42]. This stepwise progression of damage explains the clinical findings of high frequency hearing loss occurring first with ototoxic drugs.

## Conclusion

Audiologic changes have been reported in patients of MDR-TB using second line aminoglycosides which can potentially affect the communication ability of the patient. But careful audiologic monitoring may help in limiting this damage which once developed is permanent. Thus otologists and audiologists can have an important role in the management of MDR-TB in preventing the treatment related morbidity.

## Competing interests

The author(s) declare that they have no competing interests.

## Authors' contributions

DP participated in the study design, carried out audiological work and helped in drafting the manuscript. SM conceived of the study, outlined and carried out the management of the patients under study. Both the authors have read and approved the final manuscript.

## Pre-publication history

The pre-publication history for this paper can be accessed here:


